# New insights into neoadjuvant immunotherapy and chemotherapy combination for resectable N1/N2 NSCLC

**DOI:** 10.1097/JS9.0000000000003577

**Published:** 2025-09-30

**Authors:** Weiyi Jiang, Feifei Mao, Tingsu Zhang

**Affiliations:** a Department of Oncology, Ningbo Municipal Hospital of Traditional Chinese Medicine (TCM), Affiliated Hospital of Zhejiang Chinese Medical University, Ningbo, China; b Department of Thoracic Surgery, Shanghai General Hospital, Shanghai Jiao Tong University School of Medicine, Shanghai, China

*Dear Editor,*

The neoadjuvant combination of immunotherapy and chemotherapy is shown to enhance the anti-tumor immune response through synergistic effects, improving outcomes in non-small cell lung cancer (NSCLC) and contributing to reduced postoperative recurrence and prolonged survival. Yao *et al* recently reported a prospective study of 32 patients with resectable N1/N2 NSCLC who received three cycles of chemotherapy plus penpulimab followed by surgery^[1]^. The regimen achieved promising results in pathological responses, including major pathological response (MPR) and pathological complete response (pCR), as well as 1-year survival outcomes, including disease-free survival (DFS) and overall survival (OS), providing valuable data for the application of neoadjuvant immunotherapy. Nonetheless, there remains scope to further refine the study design and analysis, and such efforts would contribute to future research development. The paper follows the TITAN 2025 Guidelines on Artificial Intelligence (AI) declaration and usage^[2]^.

First, the authors adopted a single-arm study design to demonstrate the efficacy of penpulimab combined with chemotherapy as a neoadjuvant regimen. The study yielded encouraging results in terms of pathological responses and survival outcomes. However, the absence of a control group (such as direct surgery or alternative neoadjuvant regimens) somewhat limits the persuasiveness of the conclusions. The limitation also constrains a comprehensive assessment of the regimen’s relative advantages and potential drawbacks. Notably, the CheckMate 816 trial, which included resectable stage IB–IIIA NSCLC (covering both N0 and N1 disease), showed that nivolumab plus chemotherapy significantly improved pCR and EFS over chemotherapy alone, and has since been recommended by the NCCN guidelines as a standard neoadjuvant option^[3,4]^. Incorporating such comparators in future studies would allow for a more precise evaluation of the clinical value of the current approach and provide evidence of greater relevance for clinical practice.

Secondly, the difference in the proportion of adenocarcinoma and squamous cell carcinoma patients in the study still warrants further investigation. In clinical practice, adenocarcinoma usually accounts for a larger proportion of NSCLC patients, particularly among women and non-smokers^[5]^. However, the proportion of squamous cell carcinoma patients was markedly higher than that of adenocarcinoma in this study, and the authors did not provide a thorough explanation for this phenomenon. While this difference may be related to treatment responses, a more detailed discussion of patient selection criteria and their potential impact on treatment outcomes would enhance the external validity and interpretability of the results. To gain a more comprehensive understanding of the efficacy of this treatment regimen, future studies could further explore the potential impact of the distribution difference and clarify the key factors in the patient selection process.

Additionally, the table on page 28 of the study seems to have some formatting issues, with parts of the content potentially overlapping or not fully displayed, which affects the clarity of the data presentation. Although these issues did not substantively impact the main conclusions of the study, they may affect readers’ understanding and accurate interpretation of the data. It would help ensure that all data are presented clearly and accurately if the table could be appropriately adjusted in future versions.

Furthermore, although the study demonstrates the positive effects of the neoadjuvant regimen combining penpulimab and chemotherapy, the interaction between immunotherapy and chemotherapy still leaves room for further elaboration. The combined use of these two approaches is emerging as a promising strategy, and their potential mechanisms of synergy within the immune microenvironment – such as enhancing immune responses and overcoming tumor immune evasion – deserve closer examination^[6]^. If future studies could provide more mechanistic evidence in this regard, it would contribute to a more comprehensive understanding of the clinical potential of this regimen and facilitate its optimization and application.

Lastly, although the study distinguished between adenocarcinoma and squamous cell carcinoma patients, it did not perform dedicated subgroup analyses to assess differences in treatment responses with respect to MPR, pCR, DFS, and OS. Adenocarcinoma and squamous cell carcinoma differ substantially in biological characteristics, immune microenvironment, and sensitivity to immunotherapy, and these variations may significantly influence therapeutic outcomes. Future studies that conduct more systematic analyses of these histological subtypes could provide a more accurate assessment of treatment efficacy and prognosis in each subgroup, thereby offering stronger evidence to guide the optimization of individualized treatment strategies.

In summary, we highly commend the work of Yao *et al* on penpulimab plus chemotherapy as a neoadjuvant regimen for resectable N1/N2 NSCLC, which demonstrated encouraging results in pathological responses and survival outcomes, providing valuable evidence for the application of neoadjuvant immunotherapy. Nevertheless, aspects such as the inclusion of control groups, clarification of adenocarcinoma versus squamous cell carcinoma distribution, subgroup analyses, and exploration of the mechanisms underlying immunotherapy–chemotherapy synergy still warrant further refinement. Future multicenter studies with larger sample sizes will help more comprehensively evaluate treatment efficacy across different patient subgroups and provide stronger evidence for clinical application.

## Data Availability

Data sharing is not applicable to this article as no new data were generated or analyzed in this Correspondence.

## Research registration unique identifying number (UIN)

This Correspondence did not require this information.

## Declarations

AI GPT (OpenAI) was used only for grammar and sentence structure checking to ensure compliance with standard English writing conventions. The authors are fully responsible for the intellectual content.

## References

[1] Zhou D He JM Yu JC. Efficiency and safety of neoadjuvant PD-1 inhibitor (penpulimab) combined with chemotherapy in resectable N1/N2 non-small cell lung cancer: a prospective cohort study. Int J Surg 2025;111:6162–71. 40503768 10.1097/JS9.0000000000002714 PMC12430833

[2] Agha RA Mathew G Rashid R. Transparency in the reporting of artificial intelligence – the TITAN guideline. Prem J Sci 2025;10:100082.

[3] Forde PM Spicer J Lu S. Neoadjuvant nivolumab plus chemotherapy in resectable lung cancer. N Engl J Med 2022;386:1973–85. 35403841 10.1056/NEJMoa2202170 PMC9844511

[4] Riely GJ Wood DE Ettinger DS. Non-small cell lung cancer, version 4.2024, NCCN clinical practice guidelines in oncology. J Natl Compr Canc Netw 2024;22:249–74. 38754467 10.6004/jnccn.2204.0023

[5] Hou H Yu S Xu Z. Prediction of malignancy for solitary pulmonary nodules based on imaging, clinical characteristics and tumor marker levels. Eur J Cancer Prev 2021;30:382–88. 33284149 10.1097/CEJ.0000000000000637 PMC8322042

[6] Sorin M Prosty C Ghaleb L. Neoadjuvant chemoimmunotherapy for NSCLC: a systematic review and meta-analysis. JAMA Oncol 2024;10:621–33. 38512301 10.1001/jamaoncol.2024.0057 PMC10958389

